# La péritonite encapsulante sclérosante idiopathique: à propos d´un cas

**DOI:** 10.11604/pamj.2021.38.136.28043

**Published:** 2021-02-08

**Authors:** Nizar El Bouardi, Moulay Youssef Alaoui Lamrani, Meriam Haloua, Badrredine Alami, Meryem Boubou, Mustapha Maaroufi

**Affiliations:** 1 Service de Radiologie, Centre Hospitalier Universitaire Hassan II, Fès, Maroc,; 2 Faculté de Médecine et de Pharmacie, Université Sidi Mohammed Ben Abdellah, Fès, Maroc

**Keywords:** Péritonite encapsulante, imagerie médicale, tomodensitométrie, idiopathique, à propos d’un cas, Encapsulating peritonitis, medical imaging, computed tomography, idiopathic, a case report

## Abstract

La péritonite encapsulante sclérosante est une entité pathologique très rare. C´est une affection fibroinflammatoire chronique du péritoine aboutissant à la formation d´une coque péritonéale fibreuse diffuse engainant totalement ou localement les viscères abdominaux, notamment le tube digestif. Les signes cliniques sont peu spécifiques et trompeurs. L´imagerie médicale, notamment la tomodensitométrie assoie aisément le diagnostic en mettant en évidence une fine membrane péritonéale englobant un agglutinant d´anses digestives. Les formes secondaires (postdialyse péritonéale, tuberculeuse, médicamenteuse, postchimiothérapie intra-péritonéale) sont assez fréquentes, cependant la forme idiopathique reste très rare et peu de cas ont été rapporté par la littérature. Nous rapportons, à travers d´une observation, le cas d´une forme idiopathique chez une femme de 53 ans.

## Abstract (en)

Sclerosing encapsulating peritonitis is a very rare pathological entity. It is a chronic fibroinflammatory disease affecting the peritoneum and leading to the formation of diffuse egg-shell-shaped fibrous capsule which totally or locally encapsulate the abdominal viscera, in particular the digestive tract. Clinical signs are little specific and misleading. Medical imaging, including computed tomography, can help clinicians to make a diagnosis, by highlighting a thin peritoneal membrane encompassing an agglutination of digestive loops. Secondary types (postperitoneal dialysis, tuberculosis, medications, postintraperitoneal chemotherapy) are quite common, however idiopathic sclerosing encapsulating peritonitis is very rare and few cases have been reported in the literature. We here report the case of a 53-year-old woman with idiopathic sclerosing encapsulating peritonitis.

## Introduction

La péritonite encapsulante est une maladie potentiellement grave due à une encapsulation des viscères abdominaux, spécialement le tube digestif, par une membrane fibrocollagènique. Elle se produit en réponse à de multiples facteurs incitatifs avec en tête de file la dialyse péritonéale. La forme idiopathique reste exceptionnelle et de physiopathologie encore mal élucidée. Grâce aux progrès des techniques d´imagerie médicale, notamment la tomodensitométrie, cette affection se prête facilement à un diagnostic radiologique permettant un bilan préopératoire fiable. Nous rapportons un cas de forme idiopathique chez une femme de 53 ans.

## Patient et observation

Il s´agit d´une patiente de 53 ans, sans antécédent médical ou chirurgical notable, qui consulte pour un syndrome subocclusif évoluant depuis 6 mois, avec amaigrissement modérée à 3 kg. L´examen clinique a objectivé une distension abdominale modérée sans masse, ni sensibilité ou défense.

**Figure 1:**
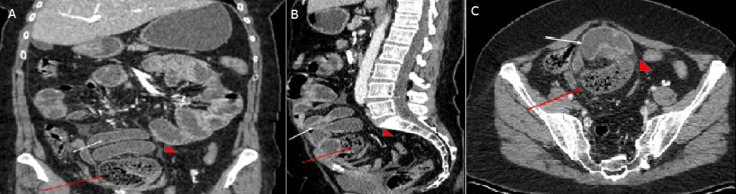
coupes coronale (A), sagittale (B) et axiale (C) de l´examen tomodensitométrique de notre patiente, acquisition après balisage digestif et après injection de produit de contraste iodé; on objective la présence d´un pseudo sac fait d´agglutinat d´anses grêliques (flèche blanche), colique (flèche rouge), entouré par une capsule péritonéale épaisse (coque fibreuse) (tête de flèche)

Le bilan biologique était sans particularités, notamment absence de syndrome inflammatoire biologique. Devant ce tableau clinique, un enteroscanner a été réalisé mettant en évidence un agglutinat d´anses digestives grêliques ainsi que le cadre colique, distendues par endroit sans zone franche de disparité de calibre, lesquels étaient englobés par une coque péritonéale épaisse formant in pseudo-sac (Figure 1). Le tout s´associait à un épanchement intra péritonéal minime.

**Figure 2:**
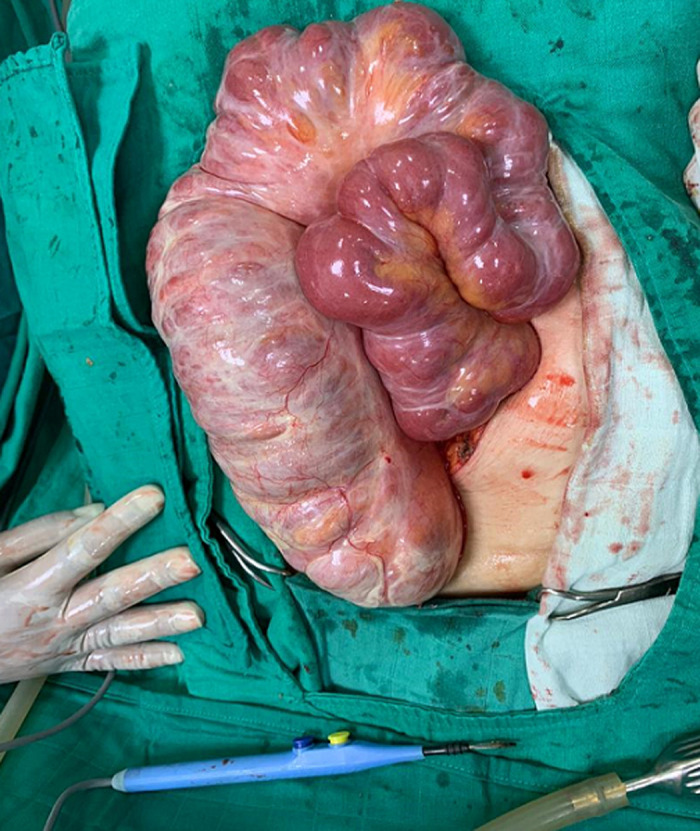
vue peropératoire mettant en évidence la coque fibreuse englobant les anses grêliques et le colon

Une laparotomie médiane à cheval sur l´ombilic a été réalisée objectivant une coque péritonéale englobant la majorité du grêle et le colon, formant une masse sacculaire (Figure 2). Le geste opératoire a consisté en des fenestrations multiples ayant permis d´extérioriser et libérer le grêle et le colon qui baignaient dans un liquide jaune citrin. Une biopsie de la coque pour étude anatomopathologique ainsi qu´un prélèvement du liquide d´ascite pour étude cytobactériologique ont était faites. L´étude du liquide péritonéal a montré un liquide inflammatoire à prédominance polynucléaires neutrophiles. Aucun germe n´a été isolé aussi bien sur examen direct que sur culture sur milieu usuels et spécifiques. L´étude anatomopathologique de la coque a montré des remaniements fibro-inflammatoires chroniques non spécifiques et n´avait pas conclu pour une cause spécifique.

Un bilan étiologique a été réalisé et est revenu négatif, et de ce fait, la forme idiopathique a été retenue. Les suites postopératoires ont été simples. L´évolution à court terme a été satisfaisante avec disparition des douleurs abdominales et reprise du transit. Mais la malade a été perdue de vue par la suite.

## Discussion

La péritonite encapsulante est une maladie rare et peu connue. Son incidence varie entre 0,7% et 7% selon la littérature. En général, elle survient vers la quatrième décade, avec une prédominance masculine [1]. C´est une maladie fibro-inflammatoire chronique du péritoine engendrant la formation d´une membrane fibreuse épaisse engainant partiellement ou totalement les organes abdominaux. Sa pathogénie est très complexe, impliquant une réactivité anormale du péritoine à un stimulus chronique, en particulier, la dialyse péritonéale. D´autre affection chroniques du péritoine y sont intriquées pouvant être d´origine infectieuses (tuberculose péritonéale, les infections gynécologiques et digestives non tuberculeuses), inflammatoires chroniques (granulomatoses rhumatismes inflammatoires chroniques), iatrogènes (chimiothérapie intra-péritonéale, usage prolongé des bêta bloqueurs), cirrhose, transplantation d´organes [2]. Les formes idiopathiques restent exceptionnelles et de physiopathologie obscure. Bien que certains auteurs impliquent la théorie de menstruations rétrogrades ou d´infections virales avec sclérose péritonéale secondaire, l´étiologie reste encore incertaine. Les symptômes cliniques sont variées et non spécifiques. Ils associent souvent une altération de l´état général, douleurs abdominales vagues et chroniques (86% des cas), des épisodes de syndromes subocclusifs avec ballonnement abdominal spontanément résolutif (82% des cas) des nausées et vomissement (54%). Parfois, elle peut se révéler en un mode aiguë soit par un syndrome occlusif franc, une ischémie voire une perforation digestive [3]. L´examen physique trouve assez souvent une masse dure de contours nets qui correspond aux anses agglutinés associée à un épanchement liquidien. Du fait que la clinique est peu spécifique, le diagnostic est souvent difficile à établir en préopératoire. Cependant, en présence de facteurs de risques dans le cadre d´un terrain prédisposant (dialyse péritonéale prolongée, une tuberculose péritonéale...) et devant un tableau clinique évocateur, le diagnostic positif pourrait être approché. Sur le plan biologique, les résultats de laboratoire ne sont pas spécifiques et liés à une infection sous-jacente, à la malnutrition et à l'inflammation. Il a été démontré que les taux de cytokines inflammatoires étaient plus élevés dans le dialysat chez les patients atteints de péritonite encapsulante que chez les témoins sous dialyse péritonéale jusqu'à des années avant le développement clinique de cette maladie [4]. Cependant, aucun biomarqueur ne s'est avéré utile pour prédire son développement. Hormis la rareté de cette entité, la sémiologie en imagerie médicale s´est nettement affiné. A cet effet, c´est la tomodensitométrie qui représente le gold standard. Les éléments du diagnostic sont assez faciles à détecter. Typiquement, on observe une agglutination des anses digestives enveloppées par une membrane épaisses formant un pseudo-sac, souvent associée à une dilatation des anses intestinales. D´autre signes inconstants peuvent être rencontrés à savoir : une infiltration et épaississement des méso, des calcifications péritonéales focales ou diffuses, une ascite cloisonnée. Elle permet d´autre part de faire un bilan préopératoire adéquat et alloue une orientation étiologique. L'imagerie par résonance magnétique reste peu exploitée pour le diagnostic, mais a probablement des rendements similaires. Les avantages comprennent l'évitement des rayonnements ionisants et une meilleure délimitation de l'enveloppe intestinale et de l'épaississement péritonéal.

L´aspect anatomopathologique de la péritonite encapsulante n´est pas spécifiques et peut chevaucher les résultats de la sclérose péritonéale simple ou de la péritonite infectieuse. Au microscope, la couche de cellules mésothéliales est dénudée associée à une prolifération de fibroblastes, dépôt de fibrocollagènique et de fibrine. Un infiltrat inflammatoire de cellules mononuclées peut être présent. La podoplanine, une glycoprotéine transmembranaire trouvée sur les cellules mésothéliales péritonéales qui se lie aux cytokines inflammatoires, aide à différencier la péritonite encapsulante sclérosante de la sclérose péritonéale et de la péritonite [5]. En général, une membrane fibrocollagènique épaissie dans le cadre du syndrome clinique décrit précédemment est suffisante pour le diagnostic.

Le faible nombre de cas rapportés explique l´absence d´un consensus thérapeutique clair. Plusieurs moyens thérapeutiques trouvent leurs indications. La corticothérapie est indiquée aux stades précoces de la maladie permettant de réduire le dépôt de fibrine et de la formation d´ascite. Le tamoxifène est utilisé pour ses propriétés antifibrotiques. Le traitement chirurgical est quant à lui indiqué dans les stades évolués de la maladie devant des épisodes de subocclusion répétitifs ou devant un abdomen aigu (occlusion, perforation, ischémie). Il consiste soit en une décortication totale qui est souvent liée à une récidive quasi constante, ou la réalisation d´incision multiples de la capsule associée à une adhésiolyse permettant de libérer les anses sténoses. La mortalité post opératoire varie entre 19 et 34% et est plus importantes chez les patients présentant un abdomen aigu.

## Conclusion

La péritonite encapsulante sclérosante reste une entité pathologique rare. La tomodensitométrie occupe une place prépondérante dans sa prise en charge permettant à la fois le diagnostic positif et un bilan pré thérapeutique adéquat. Son traitement reste essentiellement chirurgical. Malgré les progrès actuels, le pronostic reste péjoratif, avec une mortalité importante.

## Remerciements

Aux docteurs Tbaili Naima et Abdelaziz Amine pour le partage du cas et l´iconographie per opératoire.
